# Impacts of Fertilization Regimes on Arbuscular Mycorrhizal Fungal (AMF) Community Composition Were Correlated with Organic Matter Composition in Maize Rhizosphere Soil

**DOI:** 10.3389/fmicb.2016.01840

**Published:** 2016-11-16

**Authors:** Chen Zhu, Ning Ling, Junjie Guo, Min Wang, Shiwei Guo, Qirong Shen

**Affiliations:** Jiangsu Provincial Key Lab for Organic Solid Waste Utilization, Nanjing Agricultural UniversityNanjing, China

**Keywords:** fertilization regimes, organic composition, Illumina MiSeq sequencing, AMF community composition, network analysis

## Abstract

The understanding of the response of arbuscular mycorrhizal fungi (AMF) community composition to fertilization is of great significance in sustainable agriculture. However, how fertilization influences AMF diversity and composition is not well-established yet. A field experiment located in northeast China in typical black soil (Chernozem) was conducted and high-throughput sequencing approach was used to investigate the effects of different fertilizations on the variation of AMF community in the rhizosphere soil of maize crop. The results showed that AMF diversity in the maize rhizosphere was significantly altered by different fertilization regimes. As revealed by redundancy analysis, the application of organic manure was the most important factor impacting AMF community composition between samples with and without organic manure, followed by N fertilizer and P fertilizer inputs. Moreover, the organic matter composition in the rhizosphere, determined by GC–MS, was significantly altered by the organic manure amendment. Many of the chemical components displayed significant relationships with the AMF community composition according to the Mantel test, among those, 2-ethylnaphthalene explained the highest percentage (54.2%) of the variation. The relative contents of 2-ethylnaphthalene and 2, 6, 10-trimethyltetradecane had a negative correlation with *Glomus* relative abundance, while the relative content of 3-methylbiphenyl displayed a positive correlation with *Rhizophagus*. The co-occurrence patterns in treatments with and without organic manure amendment were analyzed, and more hubs were detected in the network of soils with organic manure amendment. Additionally, three operational taxonomic units (OTUs) belonging to *Glomerales* were identified as hubs in all treatments, indicating these OTUs likely occupied broad ecological niches and were always active for mediating AMF species interaction in the maize rhizosphere. Taken together, impacts of fertilization regimes on AMF community composition were correlated with organic matter composition in maize rhizosphere soil and the application of manure could activate more AMF species to interact with other species in the maize rhizosphere. This knowledge can be valuable in regulating the symbiotic system of plants and AMF, maintaining the health and high yields of crops and providing a primary basis for rational fertilization.

## Introduction

An increase in fertilizer application raises a question about how nutrient-enriched soil influences soil microbial communities. Some studies have shown that the balanced fertilization, including supplementation of nitrogen (N), phosphorus (P), and potassium (K), changed the soil microbial community and enhanced the efficient metabolism of microorganisms because of the increase in nutrient availability (Lin et al., 2012). This can lead to high soil productivity in most situations; however, excessive and inappropriate chemical fertilizer application can cause a series of environmental problems and soil degradation. In contrast to chemical fertilization, the application of organic manure to agricultural ecosystems has been used to increase soil organic matter (SOM) content and accordingly, to improve soil physical, chemical and biological qualities, which subsequently enhanced crop yield (Celik et al., 2004; Walker et al., 2004; Clemente et al., 2005). The application of organic manure can especially stimulate the microbial population and diversity (Wander et al., 1994; Zhong et al., 2010). The microorganisms are important component of soil ecosystems that characterize soil fertility (Lueders et al., 2006); thus, it is important to understand the effects of organic and inorganic fertilizer applications on soil microbial communities. For example, soil fungal diversity was found to be a good and sensitive indicator of soil fertility (He et al., 2008). The arbuscular mycorrhizal fungi (AMF) are a group of important soil organisms mediating multiple functions in agro-ecosystems.

Arbuscular mycorrhizal fungi are ancient root symbionts, and their first appearance coincides with the emergence of land plants (Bonfante and Genre, 2008). These organisms form a root symbiosis with approximately 80% of terrestrial plant species and improve nutrient and water uptake as well as pathogen resistance of their hosts in exchange for plant-assimilated carbon (Smith and Read, 2010). Therefore, it is increasingly accepted that AMF play an important role in the agro-ecosystem function. The demand of agricultural soils for certain nutrients could be reflected by changes in AMF community composition and/or diversity (Lin et al., 2012). However, how the anthropogenic disturbances drive AMF community shifts has not been examined (Rillig and Mummey, 2006; Van Der Heijden et al., 2008).

All AMF belong to the phylum *Glomeromycota* (Schüßler and Walker, 2010), and most are obligate symbionts and not culturable alone. The identification of AMF taxa in the environment is therefore not dependent on conventional culturing methods. Instead, molecular approaches (e.g., targeted polymerase chain reaction (PCR) followed by high-throughput amplicon sequencing) have become a widely used methodology for studying AMF communities (Gorzelak et al., 2012).

In this study, we used amplicon sequencing (Illumina MiSeq sequencing) to survey AMF community composition, diversity and interactions in response to organic and inorganic fertilizer applications in the rhizosphere soil of the maize crop. The objective of this study were to clarify how different fertilization regimes affect AMF community composition in the rhizosphere soil of maize crop and which was the most important key factor changing AMF community composition in the black soils.

## Materials and Methods

### Description of the Experiment

The field fertilization experiment was conducted in 2013 at the Modern Agricultural Science and Technology Demonstration Station (126°50′33″ E, 45°50′44″ N), Heilongjiang Academy of Agricultural Sciences, Harbin City, Heilongjiang Province. This region has a typical monsoon climate with an annual average temperature of 3.6°C and 486.4–543.6 mm of precipitation. The cropping regime is dominated by one maize crop per year. There were four experimental treatments: (1) NK, only mineral N and K fertilizers were applied; (2) NPK, only mineral N, P, and K fertilizer were applied; (3) NPKM, mineral N, P, and K plus organic manure was applied; and (4) M, only organic manure was applied. There were three replicates of each treatment as three independent plots with a completely randomized design. The chemical fertilizers were applied at the annual rate of 165 kg N ha^-1^, 60 kg P_2_O_5_ ha^-1^, and 75 kg K_2_O ha^-1^ as urea, superphosphate, and potassium chloride, respectively. In the NPKM treatment, the chemical fertilizers were applied at rates of 132 kg N ha^-1^, 60 kg P_2_O_5_ ha^-1^, and 75 kg K_2_O ha^-1^, respectively, plus 3,800 kg ha^-1^ of organic manures, in which the total N applied was equal to that in NPK treatment but it was divided with 20% organic N and 80% inorganic N. In the M treatment, only 19,020 kg ha^-1^ of organic manure was applied with the equal N applied to that in NPK treatment. The organic manure contained 40% moisture, 47.8% organic matter, 1.86% TN, 3.11% P_2_O_5_, and 0.85% K_2_O.

### Rhizosphere Soil Sampling and DNA Extraction

The rhizosphere soil samples were collected from each treatment in October, 2014. One composite rhizosphere sample collected from each plot consisted of the roots of five randomly selected maize plants. The roots were shaken vigorously to separate soil that was not tightly adhering to the roots. Then, 5 g of plant roots with firmly adhering soil was resuspended in 30 ml of distilled water and treated in a vortex for 1 min at high speed. The soil in the distilled water was used as the rhizosphere soil for analysis. After vacuum freeze drying, one part was stored at 4°C for GC–MS analysis, and the other part was stored at -80°C for DNA extraction.

Three rhizosphere samples from each fertilizer treatment were used for DNA extraction. The total soil DNA was extracted from 0.25 g of freeze-dried soil using a PowerSoil DNA Isolation Kit (Mo Bio Laboratories, Inc., Carlsbad, CA, USA) according to the manufacturer’s instructions. The extracted DNA was evaluated using a 1% agarose gel.

### 18S rRNA Gene Amplification and Illumina Sequencing

Polymerase chain reaction amplifications were conducted with the AMV4.5NF (5′-AAGCTCGTAGTTGAATTTCG-3′)/AMDGR (5′-CCCAACTATCCCTATTAATCAT-3′) primer set that amplifies the 18S rRNA gene. The primer set was selected in terms of the reproducibility and the ability to accurately describe AMF communities (Lumini et al., 2010). The reverse primer included a 6-bp error-correcting barcode unique to each sample. Amplicon sequencing was performed using the Illumina MiSeq platforms at Genesky Biotechnologies, Inc. (Shanghai, China).

We used the Quantitative Insights Into Microbial Ecology (QIIME) toolkit (Caporaso et al., 2010) and the UPARSE pipeline (Edgar, 2013) to treat raw high-throughput sequencing data. Barcodes and the standard primer sets were excluded. In brief, sequences below the quality score of 25 and fewer than 200 bp in length were excluded (Lin et al., 2012). Illumina MiSeq sequencing data were pretreated to remove the chimeras from the datasets. After optimizing the sequences, there were 1,214,071 valid sequences. The dominant length distribution was approximately 221–240 bp.

Then, we used the UPARSE pipeline to make an OTU table. The sequences were binned into OTUs using a 97% identity threshold, and the most abundant sequence from each OTU was selected as a representative sequence for that OTU. We used the SILVA database (SSU 119) and kmer searching^1^ to assign taxonomic data to each representative sequence. However, many fungal sequences that differed from *Glomeromycota* were obtained. Overall, 165,805 *Glomeromycota* sequences (13.5% of the total) were obtained for the primer pair AMV4.5NF/AMDGR in this study. The other major fungal groups were *Chytridiomycota*, for which 338,609 sequences (27.7% of the total) were detected, followed by *Basidiomycota*, *Zygomycota*, *Ascomycota*, and *Blastocladiomycota* (240,671 sequences, 19.7%; 105,528 sequences, 8.6%; 26,405 sequences, 2.2%; 12,249 sequences, 1.0%, respectively) (**Table 1**). Based on 97% species similarity, 42 OTUs of *Glomeromycota* were obtained out of 873 OTUs. Each sample was rarefied to the identical number of *Glomeromycota* reads (6,622) for downstream analyses. The ACE index, Shannon index and rarefaction curves were calculated by QIIME. All sequences were deposited in the NCBI Sequence Read Archive (SRA) database (accession numbers SRP078384).

**Table 1:** The proportional distributions of the sequences from each fungal phylum detected with the primer set AMV4.5NF/AMDGR for all soil samples.

	NK	NPK	NPKM	M
*Chytridiomycota*	29668 ± 2592	22614 ± 4319	22638 ± 2592	37950 ± 7747
*Glomeromycota*	9571 ± 572	7673 ± 857	13456 ± 572	24568 ± 6234
*Basidiomycota*	20064 ± 12636	20349 ± 2494	21551 ± 12636	18260 ± 5845
*Zygomycota*	13268 ± 2257	9182 ± 1736	7784 ± 2257	4942 ± 967
*Ascomycota*	982 ± 719	1097 ± 158	1332 ± 719	5390 ± 2375
*Blastocladiomycota*	807 ± 379	860 ± 193	1394 ± 379	1021 ± 294
Other Eukaryotes	17506 ± 3573	25108 ± 361	30035 ± 3573	32213 ± 4382
Unclassfied Eukaryote	762 ± 212	709 ± 65	587 ± 212	1347 ± 208

*Data expressed as mean ± SE (n = 3). NK, soil treated with chemical N and K fertilizer; NPK, soil treated with chemical N, P, and K fertilizer; NPKM, soil treated with chemical fertilizer (N, P, and K) plus organic manure; and M, soil treated with only organic manure*.

### Dissolved Organic Matter Extraction from the Rhizosphere Soil for GC–MS Analysis

Ethyl acetate-extracted dissolved organic matter (DOM) was employed in this study for GC–MS analysis. Owing to the high diversity of soil DOM, the ethyl acetate-extracted DOM does not contain all the complex soil SOM, but it provides a useful and thorough overview of likely molecular classes in DOM.

We used the approach of Song et al. (2016), which was modified by Pramanik et al. (2000) and Li et al. (2012). In brief, part of each soil sample (0.5 g) and 5 ml ethyl acetate were added to a centrifuge tube. After shaking at 170 rpm at 30°C for 1 h, the suspension was filtered (0.45 μm) into a clean centrifuge tube and was concentrated to 0.5 ml at 35°C using a vacuum rotary evaporator. The concentrated samples were then stored for GC–MS analysis.

The extracts of rhizosphere soil were analyzed by GC–MS. The initial oven temperature of 60°C was held for 3 min, increased at a rate of 5°C min^-1^ to 150°C, further increased at a rate of 3°C min^-1^ to 240°C, and then held for 10 min. The mass spectrometer was operated in the electron ionization mode at 70 eV with a source temperature of 230°C. A continuous scan from 40 to 500 m/z was used. Helium was used as the carrier gas at a linear velocity of 1.0 ml min^-1^. The mass spectra of the extracts were compared with those in the National Institute of Standards and Technology (NIST) database (Version 2.0).

### Statistical Analysis

Statistical analyses were conducted by the IMB SPSS statistical software package version 20 (IBM Corporation, New York, NY, USA). Data were analyzed with one-way ANOVA, and Duncan’s least significant difference (*P* < 0.05) was used to compare the means for each variable. Independent-samples *t*-test was performed to test significance (*P* < 0.05) of the means of ACE index and Shannon index between the treatment group with organic amendment (+M, including NPKM and M treatments) and the treatment group without organic amendment (-M, including NK and NPK treatments).

The GC–MS data were expressed as the percentage of each ethyl acetate-extracted DOM in each sample, which was calculated by dividing the sum of all peak areas per sample into its corresponding peak area. In all tests (Mantel test, Monte Carlo permutation test, Pearson correlations, and Permutational multivariate analysis), a *P*-value < 0.05 was considered statistically significant. Statistical procedures were carried out with R (version 2.15.0). Redundancy analysis (RDA), Monte Carlo permutation test and Permutational multivariate analysis were performed in R with the vegan package. In addition, we employed presence and absence matrix as the environmental factors for carrying out RDA analysis and Monte Carlo permutation test.

Mantel test was performed using the mantel function in R with the vegan package to calculate the correlation between the AMF community composition and ethyl acetate-extracted DOM composition. The Pearson correlation between the relative abundances of AMF genera and the related ethyl acetate-extracted DOM from the GC–MS analysis (*P* < 0.05 Mantel test) was determined using R.

Network analyses were carried out to better comprehend the interrelationship and interaction within the AMF community. We calculated all possible Pearson’s rank correlation coefficients via mothur (version 1.29.2) for analyzing the networks. We filtered the correlation data with a cut-off at an absolute *r*-value of 0.6 ~ 0.93, and then used a significant *P*-value of <0.05 to filter the data from the above step to improve the veracity of the networks. The networks were visualized with the interactive platform Gephi with Fruchterman–Reingold layout (Bastian et al., 2009), and a set of measures (average clustering coefficient, average path length, and modularity) was calculated (Newman, 2006).

## Results

### Effect of Fertilizations on α-Diversity of AMF Communities

**FIGURE 1:**
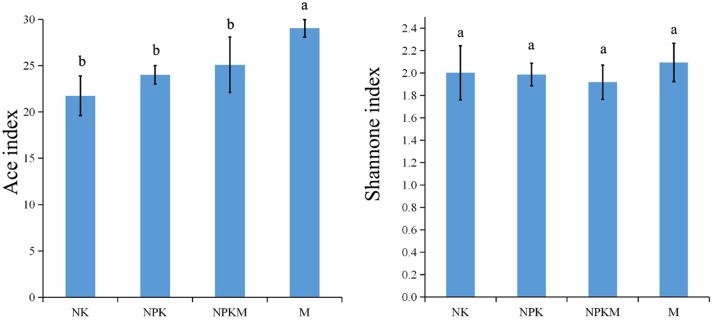
**The AMF ACE index and Shannon index for different treatments.** Bars indicated the SDs. NK, soil treated with chemical N and K fertilizer; NPK, soil treated with chemical N, P, and K fertilizer; NPKM, soil treated with chemical fertilizer (N, P, and K) plus organic manure; and M, soil treated with only organic manure. Different letters on the error bars indicate significant differences at *P* < 0.05.

The number of AMF OTUs significantly increased with the application of organic manure (M) compared to the other treatments (Supplementary Figure S1). Shannon index and ACE index were calculated based on OTU matrix of each sample in different fertilization treatments (**Figure 1**). The ACE index was significantly higher in the M treated soil.

### Effect of Fertilizations on AMF Community Composition

**FIGURE 2:**
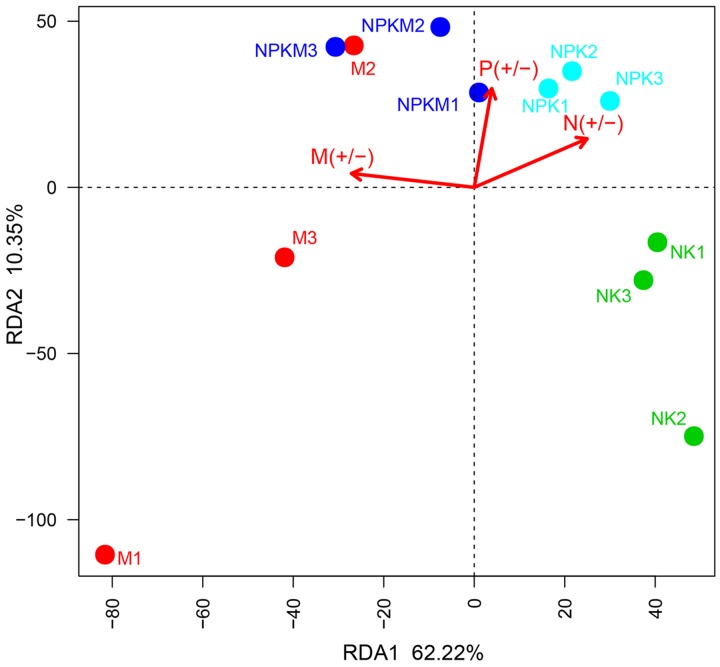
**Redundancy analysis (RDA) of the AMF community based on OTU matrix from different treatments.** The different color points represent different treatments. The three arrows represent three environment factors: M (+/-) represents treatments with (+) and without (-) organic manure; P (+/-) represents treatments with (+) and without (-) P fertilizer; N (+/-) represents treatments with (+) and without (-) N fertilizer. According to Monte Carlo permutation test the significances are: M (+/-): *r*^2^ = 0.7695, *P* = 0.002; P (+/-): *r*^2^ = 0.5048, *P* = 0.04; N (+/-): *r*^2^ = 0.6472, *P* = 0.022. NK, soil treated with chemical N and K fertilizer; NPK, soil treated with chemical N, P, and K fertilizer; NPKM, soil treated with chemical fertilizer (N, P, and K) plus organic manure; and M, soil treated with only organic manure. The number (1, 2, and 3) following the treatment indicates replicates of each treatment.

Variations in AMF community composition were clearly shown in different treatments. The first principal component explained 62.22% of the variation in the AMF community, which was predominantly sorted along the variable present or absent organic fertilization (**Figure 2**). Therefore, manure fertilization was the main reason to shift the AMF community in M and NPKM treatments compared to those in NK and NPK treatments. According to the Permutational multivariate analysis for the AMF community (Supplementary Table S1), the treatments without manure amendment (-M), including NK and NPK, were significantly (*r*^2^ = 0.41559, *P* = 0.003) different from the treatments with manure amendment (+M), including NPKM and M, in terms of community composition level. Hence, we divided the four treatments into two groups (-M and +M) for subsequent analyses.

Concomitantly, the application of organic manure was the key factor bringing about changes in AMF community composition in the maize rhizosphere and had the most explained variance (*r*^2^ = 0.7695, *P* = 0.002) with Monte Carlo permutation test (Supplementary Table S2). The second most influencing factor was N fertilizer (*r*^2^ = 0.6472, *P* = 0.022) followed by P fertilizer (*r*^2^ = 0.5048, *P* = 0.04).

### Effect of Fertilizations on Ethyl Acetate-Extracted DOM Composition of Rhizosphere

**FIGURE 3:**
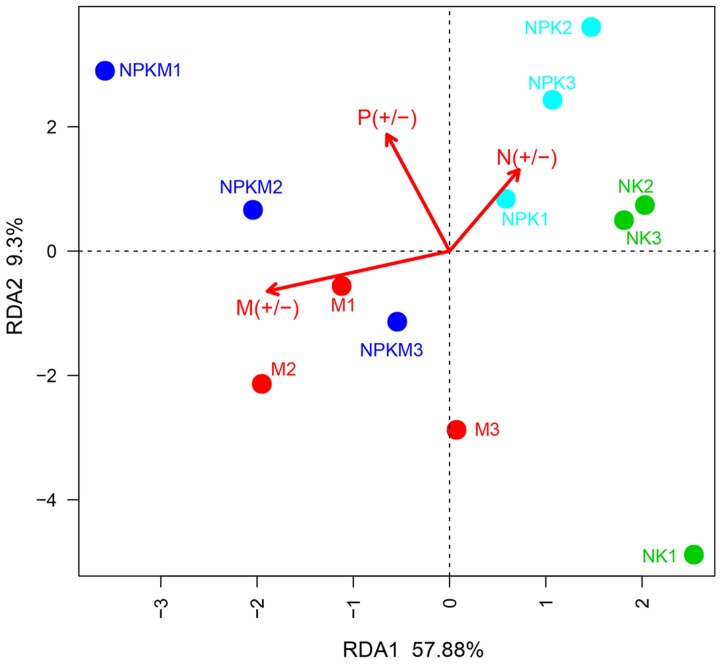
**Redundancy analysis of the identified ethyl acetate-extracted DOM.** The different fertilizer treatments for individual samples. The three arrows represent three environment factors: M (+/-) represents treatments with (+) and without (-) organic manure; P (+/-) represents treatments with (+) and without (-) P fertilizer; N (+/-) represents treatments with (+) and without (-) N fertilizer. According to Monte Carlo permutation test the significances are: M (+/-): *r*^2^ = 0.8608, *P* = 0.002; P (+/-): *r*^2^ = 0.4612, *P* = 0.73; N (+/-): *r*^2^ = 0.3676, *P* = 0.136. NK, soil treated with chemical N and K fertilizer; NPK, soil treated with chemical N, P, and K fertilizer; NPKM, soil treated with chemical fertilizer (N, P, and K) plus organic manure; and M, soil treated with only organic manure. The number (1, 2, and 3) following the treatment indicates replicates of each treatment.

According to the RDA, the first principal component explained 57.88% of the variation in ethyl acetate-extracted DOM composition. Shifts in the ethyl acetate-extracted DOM composition between the +M and -M treatments were detected, suggesting that organic manure caused the maximum changes in ethyl acetate-extracted DOM composition (**Figure 3**). The permutational multivariate analysis indicated that the chemical components in the -M treatments (NK and NPK) were significantly parted from that in the +M treatments (NPKM and M) (Supplementary Table S3). The difference in ethyl acetate-extracted DOM composition between treatments was very similar to the results of the AMF community composition. Hence, there was a potential relationship between ethyl acetate-extracted DOM composition and AMF community composition.

Similarly to AMF community composition variations, in the Monte Carlo permutation test (Supplementary Table S4), organic manure was the most significant factor influencing the ethyl acetate-extracted DOM composition (*r*^2^ = 0.8608, *P* = 0.002) while N and P fertilizers did not show significant impact. These results showed that the AMF community and the ethyl acetate-extracted DOM were similar in distribution and the application of organic manure was the key factor changing the AMF community composition and the ethyl acetate-extracted DOM composition rather than the application of chemical fertilizer.

### Relationship between AMF Community Composition and Ethyl Acetate-Extracted DOM Composition

Ethyl acetate-extracted DOM of rhizosphere soils significantly (*r* = 0.5292, *P* = 0.002) correlated with the AMF community determined by Mantel tests. In total, 59 organic compounds were identified by GC–MS (Supplementary Figure S2), among those, 11 organic compounds were identified as being significantly associated with the AMF community (**Table 2**), including 6-tert-butyl-4-methylphenol (*r* = 0.284, *P* < 0.05), pentamethyl phenyl (*r* = 0.402, *P* < 0.05), octadecanoic acid (*r* = 0.479, *P* < 0.05), 3-methylbiphenyl (*r* = 0.330, *P* < 0.05), 2-nonadecanone *O*-methyloxime (*r* = 0.390, *P* < 0.05), 1,4-dimethylnaphthalene (*r* = 0.534, *P* < 0.05), 2-ethylnaphthalene (*r* = 0.736, *P* < 0.01), octadecane (*r* = 0.344, *P* < 0.05), hexacosane (*r* = 0.358, *P* < 0.01), 2,6,10-trimethyltetradecane (*r* = 0.468, *P* < 0.01) and 3-(2, 6, 6 -trimethyl-1-cyclohexene-1-yl) allylalcohol (*r* = 0.555, *P* < 0.01). Most of the 11 related molecules were long-chain alkanes and benzenes. Meanwhile, octadecanoic acid and 3-(2, 6, 6-trimethyl-1-cyclohexene-1-yl) allylalcohol were not detected in the NPKM treatment, while 1, 4-dimethylnaphthalene was only detected in the NK treatment. However, 2-ethylnaphthalene was not detected in the NK treatment. In addition, neither octadecane nor hexacosane was detected in the +M (M and NPKM) treatments, but both were detected in the -M (NK and NPK) treatments. This showed that the application of different fertilizers drove the variation in the AMF community by changing the composition of the organic matter.

**Table 2:** Mantel correlations between the identified ethyl acetate-extracted DOM and the AMF community.

RT	Name	MV	CAS	Molecular formula	*R*
42.52	2,2′-Methylenebis(6-tert-butyl-4-methylphenol)	340	119-47-1	C_23_H_32_O_2_	0.284^∗^
13.91	Pentamethyl phenyl	148	700-12-9	C_11_H_16_	0.402^∗^
36.26	Octadecanoic acid	284	1957/11/4	C_18_H_36_O_2_	0.479^∗^
19.3	3-Methylbiphenyl	168	643-93-6	C_13_H_12_	0.330^∗^
38.41	2-Nonadecanone *O*-methyl oxime	311	36379-39-2	C_20_H_41_NO	0.390^∗^
18.04	1,4-Dimethylnaphthalene	156	571-58-4	C_12_H_12_	0.534^∗^
16.92	2-Ethylnaphthalene	156	939-27-5	C_12_H_12_	0.736^∗∗^
19.77	Octadecane	254	593-45-3	C_18_H_38_	0.344^∗^
20.84	Hexacosane	366	630-01-3	C_26_H_54_	0.358^∗∗^
22.22	2,6,10-Trimethyltetradecane	240	14905-56-7	C_17_H_36_	0.468^∗∗^
24.17	3-(2,6,6-Trimethyl-1-cyclohexene-1-yl) allyl alcohol	180	4808-01-9	C_12_H_20_O	0.555^∗∗^

*RT, retention time; MW, molecular weight of organic matter; CAS, CAS (chemical abstracts service) number, R, the correlation between a matter and AMF community; ^∗^ indicate significance at P < 0.05; ^∗∗^ indicate significance at P < 0.01*.

**FIGURE 4:**
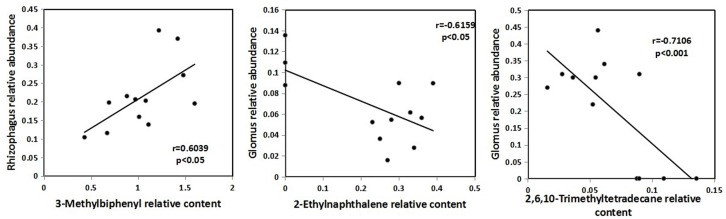
**Pearson correlations between the identified ethyl acetate-extracted DOM and AMF genera**.

Pearson correlations between the identified ethyl acetate-extracted DOM and AMF genera showed that two types of organic compounds were correlated with *Glomus* and one organic compound was correlated with *Rhizophagus* (**Figure 4**). 2-ethylnaphthalene (*r* = -0.6159, *P* < 0.05) and 2, 6, 10-trimethyltetradecane (*r* = -0.7106, *P* < 0.001) were negatively correlated with the relative abundance of *Glomus* and 3-methylbiphenyl (*r* = 0.6039, *P* < 0.05) was positively correlated with the relative abundance of *Rhizophagus*.

### Differences in AMF Co-Occurrence between Treatments with and without Organic Amendment

**FIGURE 5:**
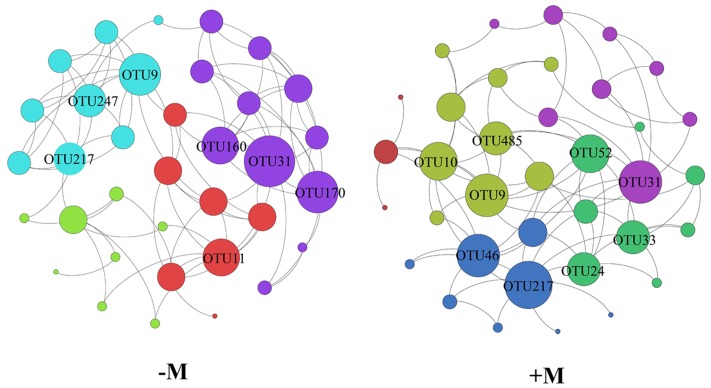
**Network analysis of the -M and +M treatments based on Pearson correlations**. The different color nodes belong to different modules. -M: the treatments without organic manure with modularity resolution of 0.540 and 4 communities; +M: the treatments with organic manure with modularity resolution of 0.438 and five communities.

**FIGURE 6:**
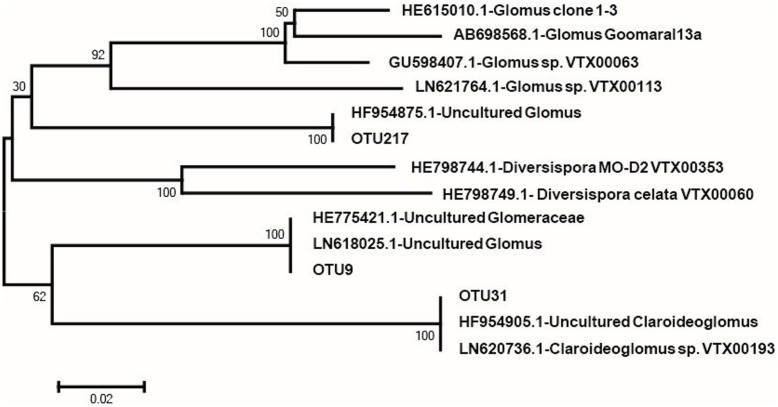
**Neighbor-joining phylogenetic tree of representative sequences of OTUs that are in both of the two networks obtained in this study and referenced sequences from the NCBI database**.

Based on the aforementioned results, we divided the four treatments into two treatments (+M and -M). Hence, we used the two treatments for network analysis. From **Figure 5**, it is apparent that the results of the network analysis were observably different between the +M treatments (NPKM and M) compared to the -M treatments (NK and NPK). In the -M treatment, the network had 36 nodes and 80 edges, and the modularity was 0.540 with four communities, whilst for the +M treatment, the network presented 35 nodes and 86 edges, and the modularity was 0.438 with five communities (**Figure 5**). Here, we arbitrarily defined the node having more than seven edges as the network hub, which was active in mediating interspecies interactions. The number of active hubs in the +M treatment was more than in the -M treatment, indicating that +M had more active species interacting with other species within the AMF community. All the hubs were classified as the order of *Glomerales* (Supplementary Table S5). In addition, more modules were found in the +M treatment. Meanwhile, three OTUs serving as hubs were shared by both networks, including OTU9, OTU31, and OTU217. It was possible that these three OTUs were active for mediating AMF species interaction and would not change their roles in the network regardless of fertilization regimes. The three OTUs belonged to different branches of the phylogenetic tree (**Figure 6**). OTU9 and OTU 217 belonged to *Glomus* and OTU31 belonged to *Claroideoglomus*.

## Discussion

The majority of sequences that were detected using the primer set AMV4.5NF/AMDGR were not from *Glomeromycota*, even though it was reported to be acceptable in several previous studies (Lumini et al., 2010; Lin et al., 2012; Dai et al., 2014; Bainard et al., 2015). We obtained nearly 170000 AMF sequences, forming 42 OTUs in all treatments. The rarefaction curves for all four treatments reached a plateau, indicating that the sequencing depth was sufficient to analyze the results (Supplementary Figure S1). In addition, the number of OTUs obtained were similar to that found in other pyrosequencing efforts from grasslands (Lumini et al., 2010; Dumbrell et al., 2011), suggesting that maize rhizosphere soils also contain abundant AMF resources compared to other ecosystems. In contrast to other reports (Oehl et al., 2005; Hijri et al., 2006; Wang et al., 2008), high-throughput sequencing data gave us a comprehensive insight into the AMF community in the agro-ecosystem in this study.

In spite of the fact that many articles have reported that the application of organic manures had negative impacts on AMF diversity (Sainz et al., 1998; Jacquot et al., 2000; Gryndler et al., 2008), several other studies have highlighted the positive influence of organic manure application on AMF population and diversity (Douds and Reider, 2003; Borie et al., 2008). Further, Albertsen et al. (2006) reported that AMF in general seemed to thrive in soil amended with organic matter. According to our results showed by ACE index and rarefaction curves (**Figure 1**; Supplementary Figure S1), the richness of AMF was increased significantly in the M treated soil, but it was not significantly different between the soils in +M and -M. The organic fertilizers have been shown to promote *Glomus* species in agricultural soils (Gryndler et al., 2006; Vestberg et al., 2011). As the soil treated with both chemical and organic fertilizers slightly, but not significantly, increased the AMF richness and diversity compared to soils with chemical-only fertilizer (**Figure 1**), these different responses of AMF to organic matter and alterations in AMF in relation to dosage of organic amendments were in accordance with Alguacil et al. (2011). Though, our results showed no significant difference in the α-diversity (ACE and Shannon indexes) between the -M and +M treatments according to the *t*-test (**Figure 1**), the AMF between-habitat diversity (β-diversity) was significantly separated between them (**Figure 2**), indicating that the changes in α-diversity and β-diversity should not occur at the same time (Van Diepen et al., 2011; Xiong et al., 2014). Some researchers reported that the change of community structure was typically correlated with shifts in functional behavior (Van Diepen et al., 2011; Fierer et al., 2013; Griffiths and Philippot, 2013). According to our results, significant difference was observed in the community structure between -M and +M treatments, but not in the richness. Therefore, these results implied that the AMF functioning probably responds first to the application of exogenous substances and the changes in microbial richness may frequently take a longer time in the maize rhizosphere soil.

P fertilizer was the main cause of changes in the soil AMF community according to the previous studies (Lin et al., 2012). However, in this study, the organic manure showed the utmost influence on the changes in AMF community composition instead of P fertilizer (**Figure 2**; Supplementary Table S2). Meanwhile, we determined that the variation in ethyl acetate-extracted DOM composition of the maize rhizosphere soils was predominantly impacted by organic amendment (**Figure 3**; Supplementary Table S4). It should be interesting to speculate that the application of organic manure was a key factor driving changes in the composition of AMF community by altering the ethyl acetate-extracted DOM composition in the maize rhizosphere soil.

Dissolved organic matter is the main substrate and energy source for microbes. The application of organic manure most likely changed the composition of the organic matter in the maize rhizosphere first, and later, the changes in organic matter composition might affect the growth and composition of AMF. There is growing evidence that the microbial composition is driven to a large extent by deterministic selection through environmental factors (Stegen et al., 2012; Valentín-Vargas et al., 2012) and the chemical nature of soil carbon drive the structure and functioning of soil microbial communities (Ng et al., 2014). Some studies have shown that AMF can directly take advantage of simple organic matter (Govindarajulu et al., 2005; Jin et al., 2005). Therefore, as biotrophic, some species of AMF that were efficient in using organic matter acquired more resources to grow and reproduce, and thereby became the dominant species after fertilization. From another perspective, root exudates are the most important sources for rhizosphere microorganisms. Yuan et al. (2016) reported that fungi out-competed bacteria in utilizing the root exudates. It is widely reported that the different fertilization regimes could change the secretion of root exudates (Carvalhais et al., 2011; Yoneyama et al., 2013; Kumar et al., 2016), which could alter the AMF community composition as well. Based on the above reasons, the AMF community composition in the maize rhizosphere soil was expectedly changed by the shift of fertilization regime, which was in accordance with the finding that organic fertilization alters the community composition of root associated fungi in *Pisum sativum* (Yu et al., 2013).

Microbes may consume or produce DOM, but the prevalent DOM composition may also (and in turn) select for a certain microbial community (Osterholz et al., 2016). It is widely supported that the specific carbon compounds trigger the growth of certain bacterial strains (Gómez-Consarnau et al., 2012), but also acknowledges the “generalists” – a functional redundancy of metabolic capabilities can lead to a minor influence of DOM quality on bacteria structure (Mou et al., 2007). In our study, the Mantel test did not allow resolving any causal relationships between AMF and ethyl acetate-extracted DOM composition; rather we provided a viewpoint of the effect of ethyl acetate-extracted DOM on AMF composition in the maize rhizosphere soil. In our study, 11 types of organic compounds were significantly correlated with AMF community composition. This indicated that not all organic matters affected the AMF community composition in the maize rhizosphere and that only some AMF were sensitive in their response to organic matter composition (**Figure 4**). Similarly, some studies (Yuan et al., 2012; Raza et al., 2015) have reported that organic compounds such as naphthyl, alkanes, and benzenes could affect the growth and spore germination of one species of fungi (*Fusarium oxysporum*).

The interaction of OTUs within habitat is one kind of internal representations of changes in AMF community structure. As caused by different fertilization regimes, the results (showed in **Figure 5**; **Table 2**) were enough to exposit that changes of the AMF community composition was correlated with the ethyl acetate-extracted DOM constituent. It is well-established that some rhizosphere compounds can regulate the interaction between microbes (van Dam and Bouwmeester, 2016; Zhu et al., 2016). Song et al. (2016) reported that ethyl acetate-extracted DOM would mediate microbial interaction in rhizosphere soils. Thus, it would be reasonable to propose that the changed constituent of ethyl acetate-extracted DOM attributed from the variation of fertilization would also lead to shifts in soil AMF co-occurrence patterns, and conducting a network analysis would be worthwhile to display the difference and similarity of AMF interactions between the soils with and without organic fertilizer (+M vs. -M). In our network analysis, the more active nodes were detected in the +M treatments than in the -M treatments (**Figure 5**). The application of organic manure activated more species of AMF involved in the maize rhizosphere soil. Soils amended with organic manure enhanced soil fertility (Chaudhry et al., 2012) and activated a more diverse group of soil microbes as compared with soils conventionally applied with inorganic fertilizers (Ai et al., 2015). Yu et al. (2013) reported that *G. mosseae* and *G. caledonium* which belonged to *Glomus* were relatively more abundant with increasing amount of the organic fertilizer applied, whereas *Paraglomus* sp. was more abundant in treatments with low dosage of organic fertilizer. The fact that different AMF had contrasting response to organic matter might indicate certain differential life strategies of AMF. Hence, some AMF species that were not active originally became active in mediating AMF species interaction after the application of organic manure. These were the major reasons of changes in AMF community composition in the maize rhizosphere with different fertilizer regimes. Only three OTUs were same in both networks: OTU9, OTU31, and OTU217. These OTUs were likely to occupy broad ecological niches and were always active for mediating AMF species interaction in the maize rhizosphere, therefore, the changes in the environment caused by fertilizations did not affect the activity of these three OTUs. In addition, they all belonged to *Glomerales* (**Figure 6**; Supplementary Table S5), which is in agreement with other studies (Oehl et al., 2003; Hijri et al., 2006; Beauregard et al., 2013) showing that *Glomerales* is widespread and is the dominant order in the soil. Moreover, at a higher phylogenetic resolution, OTU9 and OTU217 belonged to the genus *Glomus*. *Glomus* can easily survive and reproduce via mycelium, mycorrhizal spores or fragments. Therefore, *Glomus* could be more resistant and resilient to disturbances in the ecological environment thus often to play an important role in executing ecological function (Giovannetti et al., 1999; Daniell et al., 2001), such as mediating AMF species interaction.

## Conclusion

Fertilization regimes have impacts on AMF community composition and organic matter composition in the maize rhizosphere soil. A key factor in changing the AMF community composition and the organic matter composition was the application of organic fertilizer rather than chemical N and P fertilizers. The organic matter composition and the AMF community composition were significantly correlated in the maize rhizosphere. The organic amendment also resulted in the activation of more OTUs and more complex interspecific interactions in the maize rhizosphere soil, and this active role was commonly found to be played by *Glomerales*. However, to gain a better understanding of manipulating the AMF community, examinations on how and to what extent the rhizospheric DOMs impact the AMF community following the addition of soil amendments, which we predicted to impact the community in this study, should be conducted in the future.

## Author Contributions

CZ, NL, and SG proposed and organized the overall project. CZ performed the majority of the experiments. JG and MW gave assistance in lab work and laboratory analyses. CZ and NL wrote the main manuscript text. SG and QS contributed insightful discussions. All authors reviewed the manuscript.

## Conflict of Interest Statement

The authors declare that the research was conducted in the absence of any commercial or financial relationships that could be construed as a potential conflict of interest.

## Footnotes

1 http://www.mothur.org/wiki/Align.seqs
