# Supplementary material for: Impacts of Fertilization Regimes on Arbuscular Mycorrhizal Fungal (AMF) Community Composition Were Correlated with Organic Matter Composition in Maize Rhizosphere Soil
Source: Front Microbiol. 2016 Nov 16;7:1840. doi: 10.3389/fmicb.2016.01840 (PMC5110519; doi:10.3389/fmicb.2016.01840)
Supplement: Supplementary file 1 [file Data_Sheet_1.DOCX]

**Supplementary Tables and Figures for:**

# Impacts of fertilization regimes on arbuscular mycorrhizal fungal (AMF) community composition were correlated with organic matter composition in maize rhizosphere soil

Chen Zhu, Ning Ling*, Junjie Guo, Min Wang, Shiwei Guo, Qirong Shen

**Author Affiliation**

Jiangsu Provincial Key Lab for Organic Solid Waste Utilization, Nanjing Agricultural University, Nanjing, 210095, China

***Corresponding author**

Dr. Ning Ling

E-mail: [nling@njau.edu.cn](mailto:nling@njau.edu.cn)

## Supplementary data

## Table list:

**Table S1** Permutational multivariate analysis for variance of AMF community in +M treatment and -M treatment

**Table S2** Monte Carlo permutation test for RDA of AMF community

**Table S3** Permutational multivariate analysis for variance of ethyl acetate-extracted DOM in +M treatment and -M treatment

**Table S4** Monte Carlo permutation test for RDA of ethyl acetate-extracted DOM

**Table S5** Classifications of OTUs in network analysis

## Table S1 Permutational multivariate analysis for variance of AMF community in +M treatment and -M treatment

|  | Df | Sums Of Sqs | Mean Sqs | F.Model | R^2^ | Pr(>F) |
| --- | --- | --- | --- | --- | --- | --- |
| +M and -M treatment | 1 | 0.47538 | 0.47538 | 7.1113 | 0.41559 | 0.003** |
| Residuals | 10 | 0.66849 | 0.06685 |  | 0.58441 |  |
| Total | 11 | 1.14387 | 1 |  |  |  |

**indicate significant correlations (*P* < 0.01), *P* values based on 999 permutations

**Table S2** Monte Carlo permutation test for RDA of AMF community

|  | RDA1 | RDA2 | r^2^ | P |
| --- | --- | --- | --- | --- |
| M(+/-) | -0.97685 | 0.21394 | 0.7695 | 0.002** |
| P(+/-) | -0.00235 | 1 | 0.5048 | 0.04* |
| N(+/-) | 0.97332 | 0.22946 | 0.6472 | 0.022* |

*indicate significant correlations (*P* < 0.05); **indicate significant correlations (*P* < 0.01), *P* values based on 999 permutations**Table S3** Permutational multivariate analysis for variance of ethyl acetate-extracted DOM in +M treatment and -M treatment

|  | Df | Sums Of Sqs | Mean Sqs | F.Model | R^2^ | Pr(>F) |
| --- | --- | --- | --- | --- | --- | --- |
| +M and -M treatment | 1 | 0.13193 | 0.13193 | 10.643 | 0.51558 | 0.004** |
| Residuals | 10 | 0.12395 | 0.12395 |  | 0.48442 |  |
| Total | 11 | 0.25588 | 1 |  |  |  |

**indicate significant correlations (*P*<0.01), P values based on 999 permutations

**Table S4** Monte Carlo permutation test for RDA of ethyl acetate-extracted DOM

|  | RDA1 | RDA2 | r^2^ | P |
| --- | --- | --- | --- | --- |
| M(+/-) | -0.95536 | -0.29543 | 0.8608 | 0.002** |
| P(+/-) | -0.38127 | 0.92446 | 0.4612 | 0.073 |
| N(+/-) | 0.71519 | 0.69893 | 0.3676 | 0.136 |

**indicate significant correlations (*P*<0.01), *P* values based on 999 permutations.

**Table S5** Classifications of OTUs in network analysis

|  | Phylum | Class | Order | Family | Genus |
| --- | --- | --- | --- | --- | --- |
| **OTU9** | ***Glomeromycota*** | ***Glomeromycetes*** | ***G****lomer****ales*** | **Unclassified** | **Unclassified** |
| OTU10 | *Glomeromycota* | *Glomeromycetes* | *Glomerales* | Unclassified | Unclassified |
| OTU11 | *Glomeromycota* | *Glomeromycetes* | *Glomerales* | *Glomeraceae* | *Septoglomus* |
| OTU24 | *Glomeromycota* | *Glomeromycetes* | *Glomerales* | Unclassified | Unclassified |
| **OTU31** | ***Glomeromycota*** | ***Glomeromycetes*** | ***Glomerales*** | ***Glomeraceae*** | ***Glomus*** |
| OTU33 | *Glomeromycota* | *Glomeromycetes* | *Glomerales* | *Glomeraceae* | Unclassified |
| OTU46 | *Glomeromycota* | *Glomeromycetes* | *Glomerales* | Unclassified | Unclassified |
| OTU52 | *Glomeromycota* | *Glomeromycetes* | *Glomerales* | Unclassified | Unclassified |
| OTU160 | *Glomeromycota* | *Glomeromycetes* | *Glomerales* | Unclassified | Unclassified |
| OTU170 | *Glomeromycota* | *Glomeromycetes* | *Glomerales* | *Glomeraceae* | *Septoglomus* |
| **OTU217** | ***Glomeromycota*** | ***Glomeromycetes*** | ***Glomerales*** | **Unclassified** | **Unclassified** |
| OTU247 | *Glomeromycota* | *Glomeromycetes* | *Glomerales* | Unclassified | Unclassified |
| OTU485 | *Glomeromycota* | *Glomeromycetes* | *Glomerales* | Unclassified | Unclassified |

The OTUs in bold indicate they were in both networks.

## Figure list:

**Fig. S1** Rarefaction curves of the observed OTUs of AMF. Bars indicated the SEs. NK: soil treated with chemical N and K fertilizer; NPK: soil treated with chemical N, P and K fertilizer; NPKM: soil treated with chemical fertilizer (N, P and K) plus organic manure; and M: soil treated with only organic manure.

**Fig. S2** GC-MS chromatogram of ethyl acetate-dissolved organic matter from maize rhizosphere soil. NK: soil treated with chemical N and K fertilizer; NPK: soil treated with chemical N, P and K fertilizer; NPKM: soil treated with chemical fertilizer (N, P and K) plus organic manure; and M: soil treated with only organic manure.


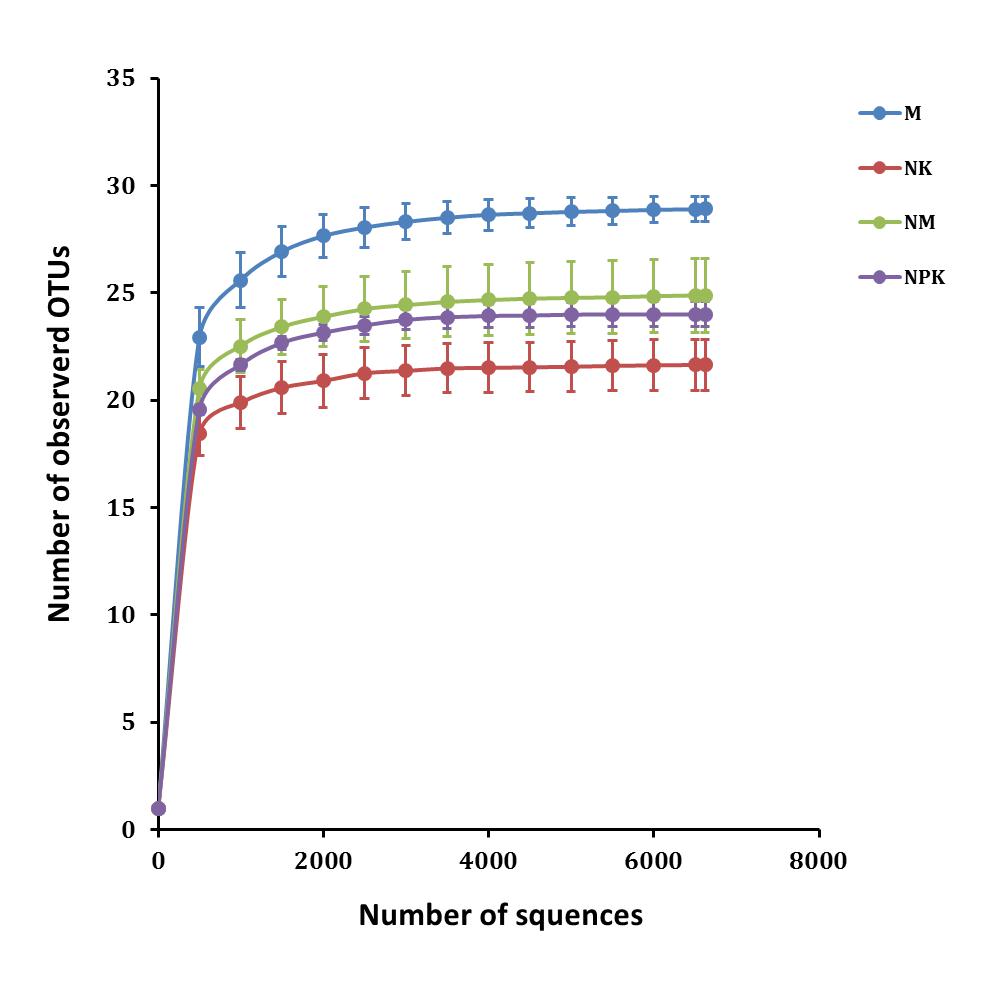


**Fig. S1** Rarefaction curves of the observed OTUs of AMF. Bars indicated the SEs. NK: soil treated with chemical N and K fertilizer; NPK: soil treated with chemical N, P and K fertilizer; NPKM: soil treated with chemical fertilizer (N, P and K) plus organic manure; and M: soil treated with only organic manure.


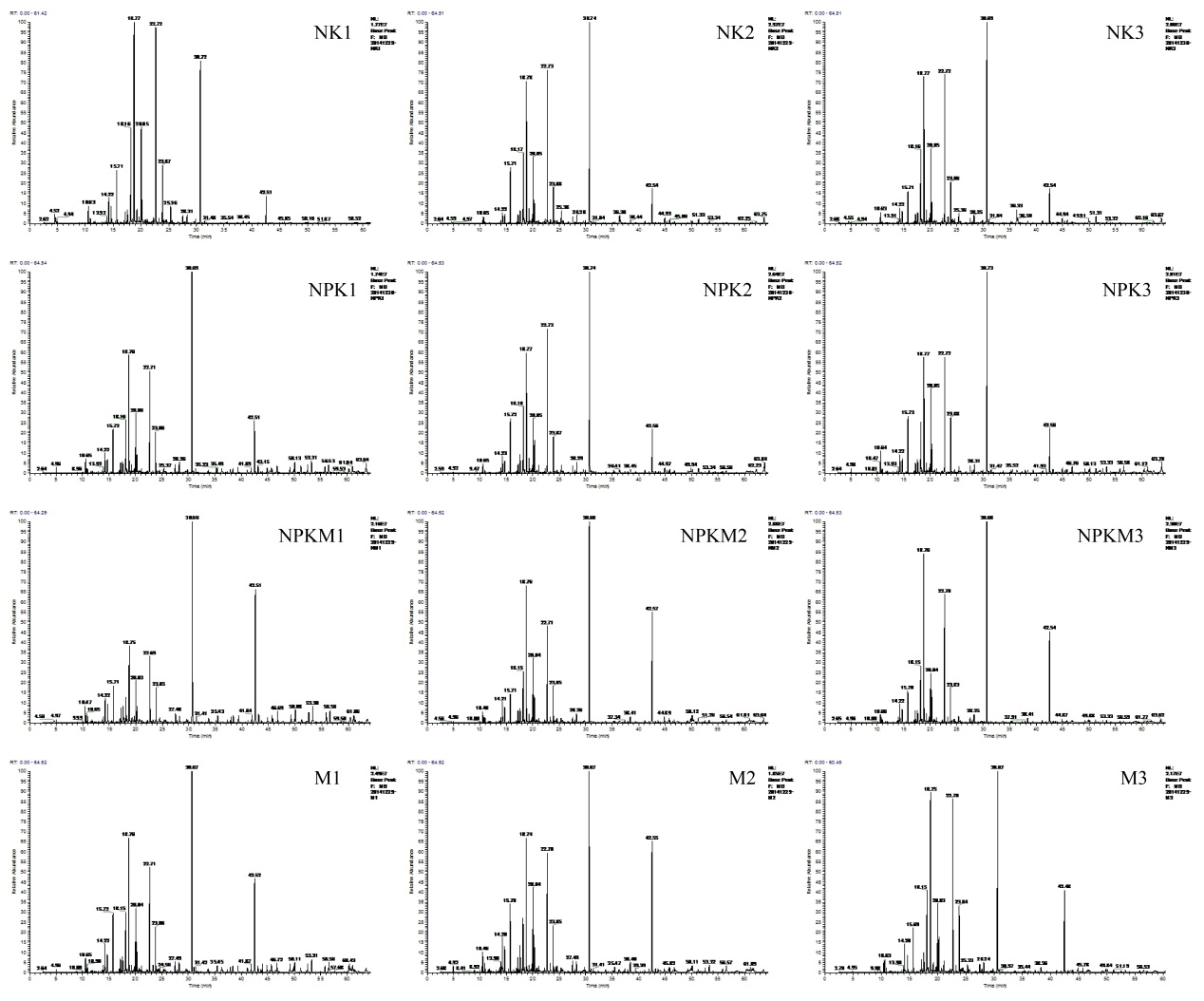


**Fig. S2** GC-MS chromatogram of ethyl acetate-extracted DOM from maize rhizosphere soil. NK: soil treated with chemical N and K fertilizer; NPK: soil treated with chemical N, P and K fertilizer; NPKM: soil treated with chemical fertilizer (N, P and K) plus organic manure; and M: soil treated with only organic manure.
